# Conventional craniotomy versus conservative treatment in patients with minor spontaneous intracerebral hemorrhage in the basal ganglia

**DOI:** 10.1186/s41016-022-00288-y

**Published:** 2022-08-19

**Authors:** Ning Wang, Weiwei Lin, Xuanhao Zhu, Qi Tu, Daqian Zhu, Shuai Qu, Jianjing Yang, Linhui Ruan, Qichuan Zhuge

**Affiliations:** 1 Brain Center, Affiliated Zhejiang Hospital, Zhejiang University School of Medicine, Hangzhou, 310000 China; 2 Department of Neurosurgery, Second Affiliated Hospital of Zhejiang University School of Medicine, Zhejiang University, 88 Jiefang Road, Hangzhou, 310009 Zhejiang China; 3 Zhejiang Provincial Key Laboratory of Aging and Neurological Disorder Research, The First Affiliated Hospital of Wenzhou Medical University, Wenzhou, 325000 China; 4 Department of Neurosurgery, The First Affiliated Hospital of Wenzhou Medical University, Wenzhou, 325000 Zhejiang China

**Keywords:** Basal ganglia, Conservative treatment, Craniotomy, Intracerebral hemorrhage

## Abstract

**Background:**

The treatment for spontaneous intracerebral hemorrhage (ICH) is still controversial, especially for hematomas in the basal ganglia. A retrospective case-control study with propensity score matching was performed to compare the outcomes of conventional craniotomy and conservative treatment for patients with minor ICH in the basal ganglia.

**Methods:**

We retrospectively collected the data of consecutive patients with minor basal ganglia hemorrhage from January 2018 to August 2019. We compared clinical outcomes of two groups using propensity score matching. The extended Glasgow outcome scale obtained by phone interviews based on questionnaires at a 12-month follow-up was used as the primary outcome measure. According to a previous prognosis algorithm, patients were divided into good and poor prognosis groups to obtain a dichotomized (favorable or unfavorable) outcome as the primary outcome. Secondary outcomes included hospitalized complications, mortality, and modified Rankin score at 12 months.

**Results:**

A total of 54 patients were analyzed, and the baseline characteristics of patients in the surgery and conservative treatment groups were well matched. The primary favorable outcome at 12 months was significantly higher in the conservative treatment group than in the surgery group (81% vs 44%; OR 1.833, 95% CI 1.159–2.900; *P*=0.005). The incidence of pneumonia in the surgery group was significantly higher than that in the conservative treatment group (*P*=0.005).

**Conclusions:**

It is not recommended to undertake conventional craniotomy for patients with a minor hematoma (25–40 ml) in the basal ganglia. An open craniotomy might induce worse long-term functional outcomes than the conservative treatment.

## Background

Spontaneous intracerebral hemorrhage (ICH) remains a significant cause of global morbidity and mortality. It affects 0.50–1.1 million patients in China each year [1], and the median 1-month case mortality is 40%, among which many survivors remain severely disabled [2]. The most common location of ICH is the basal ganglia [3]. However, despite the high frequency of ICH within the basal ganglia, its treatment strategy remains controversial among neurosurgeons and neurologists [4].

Surgery has the potential to improve neurological recovery of ICH because early mass removal might reduce nervous tissue damage, possibly by relieving local ischemia [5–7] or the removal of noxious chemicals [8, 9]. Nonetheless, the effect of surgery does not seem to be homogeneous, with expert opinion, mechanistic reasoning, and trial data all indicating that early surgery benefits only some particular clots. Thus, most neurosurgeons would not remove a small ICH in the internal capsule or basal ganglia because its surgical approach path is too close to the motion-conducting regions.

Several prospective randomized controlled trials (RCTs) were undertaken during the previous four decades to compare the outcome of early surgery with conservative treatment, the results of which were neutral [10–15]. The results of a subgroup analysis from the International Surgical Trial in Intracerebral Hemorrhage (STICH) indicated that patients with spontaneous ICH in the basal ganglia show no overall advantage from early surgery when compared with initial conservative treatment [16]. However, these trials had enrolled only small numbers of patients from China. A randomized controlled trial in China demonstrated improvements in independent survival in minimally invasive surgically treated patients with small basal ganglion hemorrhages [17].

Traditional craniotomy is generally used to remove hematomas. A large flap is created, and the brain is exposed, detached, and manipulated to inspect the site of bleeding, and the blood can be suctioned and hemostasis applied to multiple areas [18]. With the development of neurosurgery and the updating of surgical instruments, the efficacy of craniotomy is worth investigating. The aim of this trial was to determine whether craniotomy evacuation would improve outcomes compared with conservative treatment in patients with small basal ganglion intracerebral hemorrhages (volume ≤40 ml) in China.

## Methods

### Patient selection

Consecutive patients with acute spontaneous ICH (<24 h) were retrospectively enrolled from the First Affiliated Hospital of Wenzhou Medical University, Zhejiang Province, China, between January 2018 and August 2019. A diagnosis of ICH was confirmed by computerized tomography (CT) scan of the head and was made according to the diagnostic criteria of the guidelines for the management of ICH [19].

Patients were eligible if (1) they had a diagnosis of ICH and treated in the neurosurgery department; (2) their hematoma was located in the basal ganglia (internal or external capsule, caudate nucleus, putamen, or more than one of the abovementioned structures); (3) the hematoma volume was between 25 and 40 mL; (4) 40 ≤ age ≤75 years; and (5) the patients reached the hospital within 24 h of ictus, whereas they were ineligible if (1) the hemorrhage was induced by an aneurysm, angiographically proven arteriovenous malformation, infarction, trauma, or tumor; (2) the hemorrhage originated from the cerebellum, brainstem, or lobes; (3) they had severe pre-existing physical or mental disabilities or comorbidities that could interfere with the assessment of their outcomes; (4) they had coagulation disorders; (5) they had a prior history of stroke with neurological deficits; or (6) patients who needed urgent evacuation because of a hernia.

### Baseline parameters and treatment procedure

Demographics, medical history, medication, the time between ictus and arrival at the emergency department, and baseline clinical and radiological information were obtained from the emergency and hospitalization datasets. The baseline volume of ICH was calculated by the formula A × B × C/2 according to the CT scan [20]. Intraventricular hemorrhage (IVH) was not included in the volume calculated. The patient’s conscious disturbance and neurological function were evaluated through the Glasgow coma score (GCS) and modified Rankin score (mRS) at the time of admission.

Patients were divided into a surgery group and a conservative treatment group according to the treatment they received. For patients in the surgery group, the hematoma was carefully evacuated under direct vision through a microscope via a small penetration in the superior temporal gyrus or other noneloquent area. Decompressive craniotomy was performed if necessary. In the conservative treatment group, no invasive procedures were applied. All patients were given the best medical treatment guided by the International Recommendations [19] and if necessary, they were individualized for particular patients.

### Outcomes

The primary outcome was a prognosis-based favorable or unfavorable outcome dichotomized from the Extended Glasgow Outcome Scale (GOSE) at 12 months after ictus. GOSE was evaluated from the answers to 14 questions in a questionnaire completed by the patients or their relatives [21]. The questionnaires were completed by telephone interviews conducted by independent, blinded to the treatment group interviewers.

The prognostic outcome was designed to be different types of outcomes with two different levels for the patient’s status [22]. Prognosis was estimated using the following algorithm, which had been developed from patients with a broad range of different ICH:

$$\mathrm{Prognostic}\ \mathrm{score}=10\times \mathrm{GCS}-\mathrm{age}-0.64\times \mathrm{volume}$$

Patients were divided into good and poor prognosis groups using the predefined cutoff of 27.672 for supratentorial intracerebral hemorrhage [16]. For patients with a poor prognosis, the outcome was regarded as favorable if GOSE was better than upper severe disability, while for patients in the good prognosis group, favorable outcomes included a good recovery and moderate disability.

Secondary outcomes included the incidence of hospitalized complications, mortality, and prognosis-based dichotomized mRS at 12 months of follow-up. For the good prognosis group, a Rankin score of 0–2 was judged as a favorable outcome, while for those with a poor prognosis, the equivalent thresholds were three for the Rankin score. Death was stratified as an unfavorable outcome for all patients.

### Statistical analysis

Matching analysis was performed on the basis of the estimated propensity scores of observed clinical factors that may influence decision making for treatment strategy, that is, the patient’s age, GCS, volume of hematoma, and midline shift. Patients in the two groups were one-to-one matched for the similarity of their propensity score by nearest neighbor matching. The standardized mean difference (SMD) was calculated to assess the balance detail after propensity score match (PSM). The SMD less than 0.2 was defined as acceptable matching and all the variables achieved (Table 1).

**Table 1:** The variables comparison before and after PSM

	Before matching (*n*=87)				After matching (*n*=54)			
	Surgery group (*n*=45)	Conservative treatment (*n*=41)	SMD	*P* value	Surgery group (*n*=27)	Conservative treatment (*n*=27)	SMD	*P* value
Age	52.6 (8.34)	56.83 (9.66)	0.469	0.032	54.9 (9.60)	54.9 (8.64)	0.004	0.988
GCS	9.38 (2.73)	11.34 (2.55)	0.744	0.001	10.3 (2.48)	10.6 (2.45)	0.120	0.661
Midline shift	6.81 (2.33)	5.59 (1.92)	0.572	0.010	5.9 (2.06)	5.8 (1.75)	0.031	0.91
Volume	30.03 (3.14)	29.74 (4.26)	0.077	0.722	29.9 (3.07)	30.2 (4.46)	0.073	0.790

*PSM*, Propensity score match, *SMD* Standardized mean difference, *GCS*, Glasgow Coma Score

Data for categorical variables were reported as the number and percentage in each group. The percentage was reported to no decimal places. For continuous variables, the mean and SD, as well as the median, quartiles, maximum, and minimum were calculated. Quantitative data were analyzed by Student’s *t* test or the Mann-Whitney test depending on the distribution. Categorical data were compared by Pearson’s chi-squared test or continuous corrected *χ*^2^ tests where appropriate. Outcomes were reported as odds ratios (OR) with 95% CI.

The primary outcome was a simple categorical variable compared by chi-square tests for prognosis-based favorable and unfavorable outcomes on GOSE. After adjusting for the covariates GCS, volume of the hematoma, and midline shift, multivariate-adjusted binary logistic regression was performed to calculate the OR and 95% CI for the primary outcome. Secondary analysis consisted of *χ*^2^ tests for mortality and 12-month prognosis-based mRS.

Prespecified subgroup analyses were also undertaken for age (<55, ≥55), volume of the hematoma (≤30, >30), midline shift (≤6, >6), GCS (5–8; 9–12; 13–15), IVH, status of the worse limb (weak, paralyzed), and two prognosis groups (good and poor). *P* values were reported to three decimal places or at *P* <0.0001. In this study, the statistical significance was defined as a two-tailed *P* value less than 0.05 (*P* < 0.05). The analyses were performed with SPSS 23.0 (IBM Corp.), the Stata Statistical Software Release 12.0 (College Station, TX, StataCorp LP), and R 4.1.3.

## Results

**Fig. 1:**
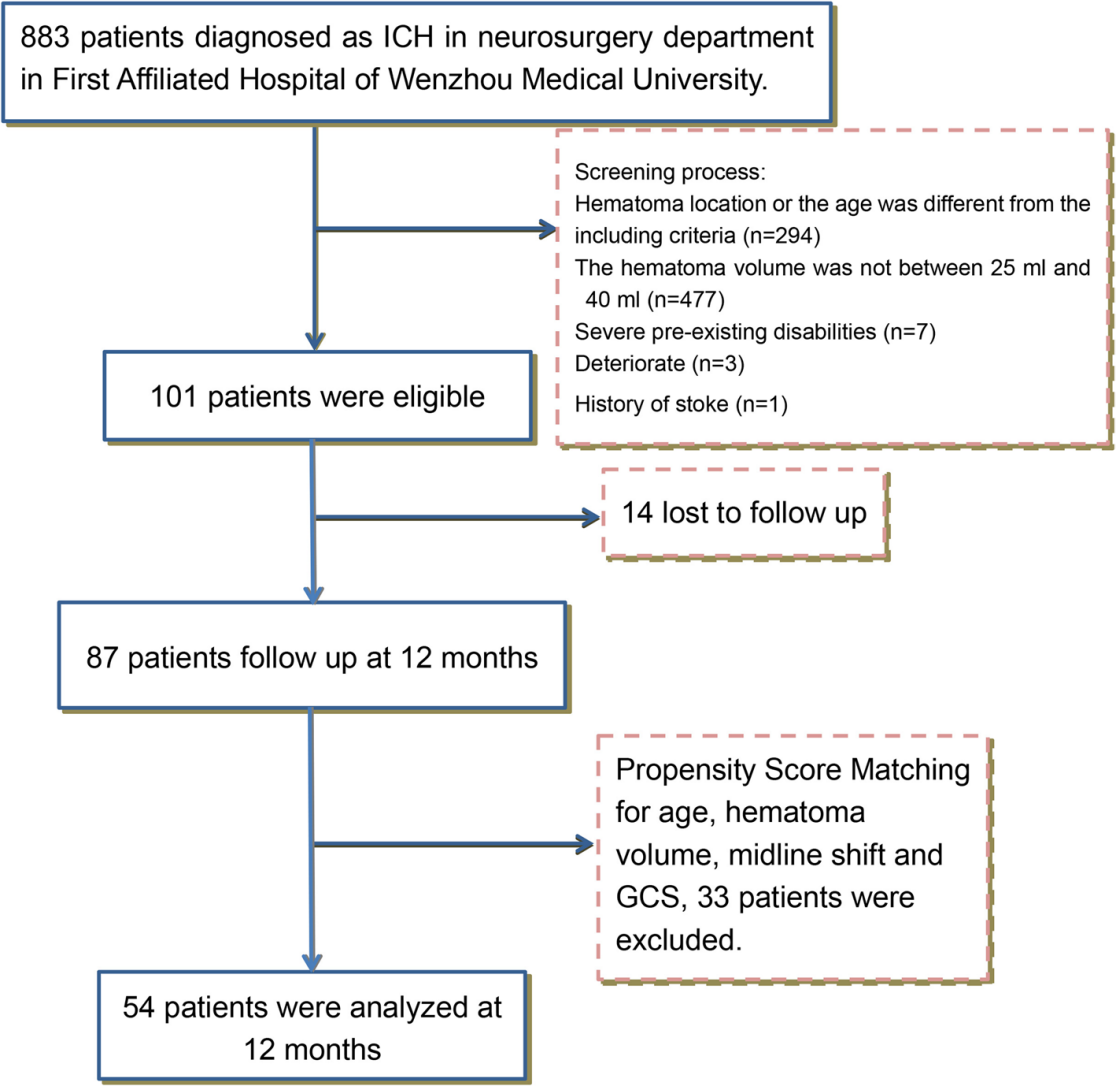
Study profile. ICH, intracerebral hemorrhage; GCS, Glasgow Coma Score

A total of 101 patients were eligible in the First Affiliated Hospital of Wenzhou Medical University. Fourteen patients were lost to follow-up, and 33 patients were excluded after the propensity score matching (PSM) for age, GCS, hematoma volume, and midline shift. This analysis, therefore, includes 54 patients, of whom 27 patients underwent evacuation (Fig. 1). Most patients were followed-up for 12 months, except for five patients who were 1 month short. Patients’ baseline characteristics are shown in Table 1, including age, sex, neurological status, and previous medical history. The two groups seemed to be well-matched at baseline, 78% were men, and their age was between 40 and 75 years (mean 54.87±9.05 years). At admission, 26% of patients in the surgery group and 22% of patients in the conservative treatment group were in a coma (GCS ≤8) and only 22% of patients had a GCS ≥13. Paralysis of the affected limb happened in most patients in both groups (93% and 81%, respectively) (Table 2). The details of the hematoma are shown in Table 3. The median volume of the hematoma was 29.5 mL (26.3–32.8 mL), and the midline shift was 5.85±1.89 mm. Ten (19%) patients had concurrent intraventricular hemorrhage. Table 4 shows the comparison of the hospitalized complications between the two groups. There was no significant difference in the overall complication rate between the two groups. Exceptionally, the incidence of pneumonia in the surgery group was significantly higher than that in the conservative treatment group (*P*=0.005). At 12 months, epileptic seizures had affected one patient in the conservative treatment group and 2 in the surgery group, one of whom had an intracerebral ischemic stroke at the same time.

**Table 2:** Baseline characteristics of the patients

	Surgery group (*n*=27)	Conservative treatment (*n*=27)	*P* value
Age (years)			0.897^*^
Median (IQR; range)	51 (47,61; 41–75)	55 (49,62; 40–74)	
Sex			0.190^†^
Male	19 (70%)	23 (85%)	
Female	8 (30%)	4 (15%)	
Time between ictus and emergency room			0.156^*^
Median (IQR; range)	3.0 (2.0,5.0; 1–8)	4.0 (3.0,7.0; 1–23)	
Glasgow Coma Score			0.946^†^
5–8	7 (26%)	6 (22%)	
9–12	14 (52%)	15 (56%)	
13–15	6 (22%)	6 (22%)	
mRS			0.129^†^
4	22 (82%)	17 (63%)	
5	5 (18%)	10 (37%)	
Localizing arm			0.418^†^
Weak	2 (7%)	5 (19%)	
Paralyzed	25 (93%)	22 (81%)	
Localizing leg			0.715^†^
Weak	5 (18%)	4 (15%)	
Paralyzed	22 (82%)	23 (85%)	
Medical history			
Hypertension	22 (82%)	19 (70%)	0.340^†^
On antihypertensive drugs	19 (70%)	16 (59%)	0.393^†^
Diabetes mellitus	2 (7%)	4 (15%)	0.665^‡^
Previous myocardial infarction	0 (0%)	1 (4%)	1.000^‡^
Previous stroke	1 (4%)	2 (7%)	1.000^‡^
Prognostic score			0.715^§^
Mean (SD)	28.9 (27.37)	31.8 (29.12)	
Prognostic score category			1.000^†^
Poor	13 (48%)	13 (48%)	
Good	14 (52%)	14 (52%)	

For continuous variables, data are median (IQR; range) and mean (SD); for categorical variables, data are number (%). *mRS*, Modified Rankin Score, *IQR*, Interquartile range, *SD*, Standard deviation

^†^*χ*^2^ test; ^‡^Continuous corrected *χ*^2^ test; ^*^Mann-Whitney test; ^§^Student’s *t* test

**Table 3:** Characteristics of the hematomas

	Surgery group (*n*=27)	Conservation treatment group (*n*=27)	*P* value
Volume (ml)			0.790^§^
Median (IQR; range)	29.6 (27.0,32.4; 25.4–36.6)	28.5 (26.3,33.7; 25.3–37.6)	
Mean (SD)	29.9 (3.07)	30.2 (4.46)	
IVH	5 (19%)	5 (19%)	1.000^†^
Midline shift (mm)			0.910^§^
Median (IQR; range)	5.5 (4.5,7.4; 0–10.19)	5.6 (4.6,7.4; 1.8–8.8)	
Mean (SD)	5.9 (2.06)	5.8 (1.75)	
Side of hemorrhage			0.785^†^
Left	13 (48%)	14 (52%)	
Right	14 (52%)	13 (48%)	

For continuous variables, data are median (IQR; range) and mean (SD); for categorical variables, data are number (%). *IVH*, Intraventricular hemorrhage, *IQR*, Interquartile range, *SD*, Standard deviation

^†^*χ*^2^ test; ^§^Student’s *t* test

**Table 4:** Hospitalized complications

Category	Surgery (*n*=27)	Conservative treatment (*n*=27)	*P* value
Complications	20 (74%)	15 (56%)	0.154^†^
Pneumonia	9 (33%)	1 (4%)	0.005^†^
Urinary infection	2 (7%)	4 (15%)	0.665^‡^
Gastrointestinal bleeding	1 (4%)	3 (11%)	0.603^‡^
Liver dysfunction	6 (22%)	10 (37%)	0.233^†^
Renal dysfunction	4 (15%)	2 (7%)	0.665^‡^
Venous thrombus	3 (11%)	4 (15%)	1.000^‡^
Others	2 (7%)	1 (4%)	1.000^‡^

Data are number or number (%)

^†^*χ*^2^ test; ^‡^Continuous corrected *χ*^2^ test

With the prognosis-based dichotomy of GOSE, twelve (44%) of 27 patients in the surgery group had a favorable outcome at 12 months compared with 22 (81%) of 27 patients in the conservative treatment group (Table 5; OR 1.833, 95% CI 1.159–2.900; *P*=0.005).

**Table 5:** Prespecified outcomes at 12 months

	Surgery group (*n*=27)	Conservative treatment group (*n*=27)	*P* value	OR (95% CI)
Primary outcome				
Prognosis based			0.005^†^	1.833 (1.159–2.900)
Unfavorable	15 (56%)	5 (19%)	..	
Favorable	12 (44%)	22 (81%)	..	
Secondary outcomes				
Mortality			0.119^‡^	1.174 (1.003–1.374)
Dead	4 (15%)	0	..	
Alive	23 (85%)	27 (100%)	..	
Prognosis based modified Rankin			0.006^†^	2.000 (1.165–3.432)
Unfavorable	17 (63%)	7 (26%)	..	
Favorable	10 (37%)	20 (74%)	..	

Data are number or number (%)

^†^*χ*^2^ test; ^‡^Continuous corrected *χ*^2^ test

The mortality rate at 12 months tended to be lower in the conservative group (absolute difference 14.8%; OR 1.174, 95% CI 1.003–1.374; *P*= 0.119). Three (11%) of 27 patients died within 30 days in the surgery group, one of which had rebleeding after the first evacuation and his relatives refused permission for a second operation, one developed severe renal failure and the other one died for an unknown reason.

The prognosis-based modified Rankin score showed favorable outcomes in 10 (37%) of 27 patients in the surgery group, which was significantly lower than the favorable outcomes in the conservative treatment group (*P*= 0.006; Table 5).

**Fig. 2:**
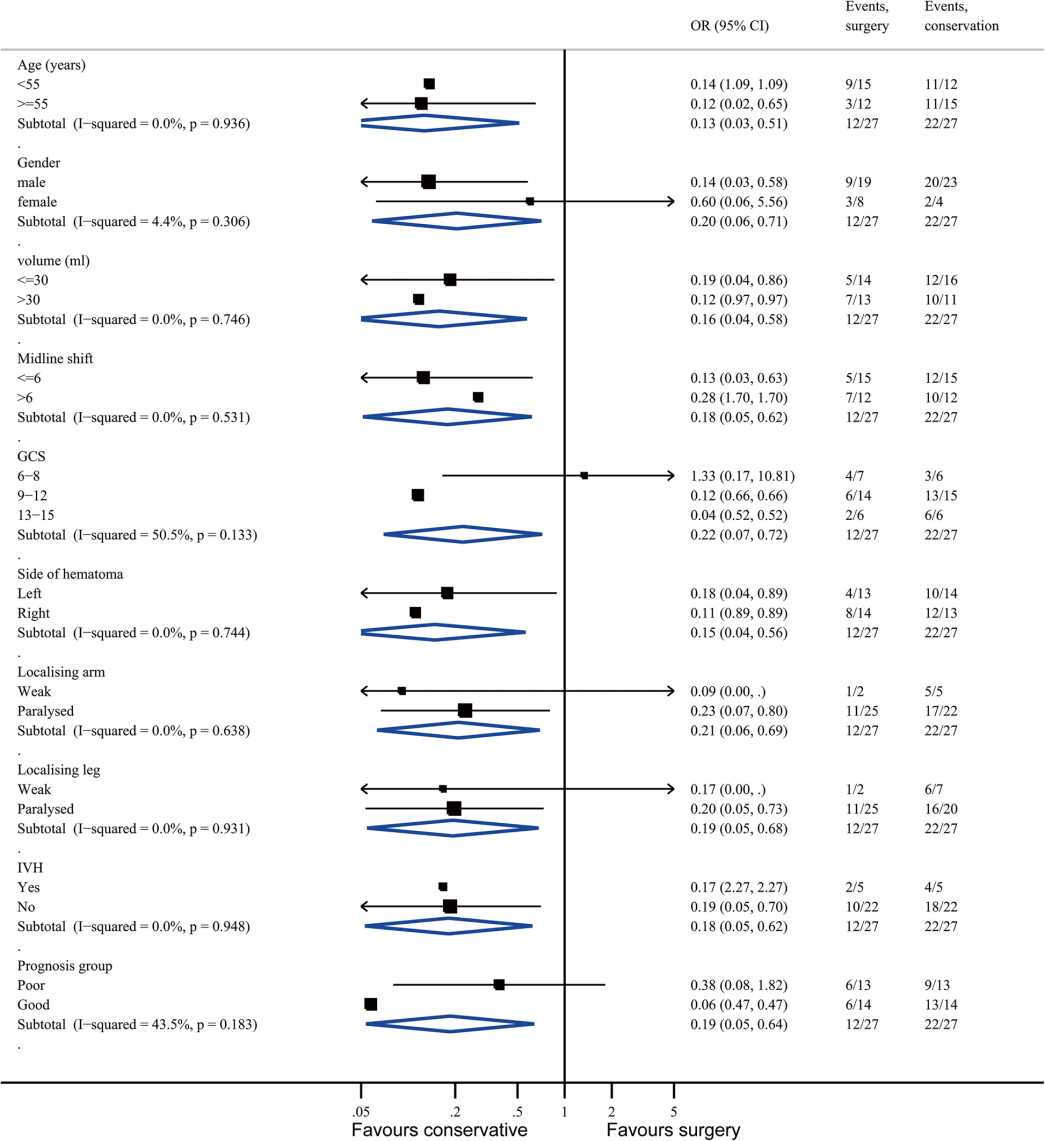
Subgroup analysis forest plot. GCS, Glasgow Coma Score

The prespecified subgroup analyses are shown in Fig. 2, none of which showed heterogeneity of treatment response. At 12 months, 2 (8%) of 26 patients died in the poor prognosis group and 9 (35%) had severe lower disability; 2 (7%) of the 28 patients in the good prognosis group died; and 2 (7%) were severely lower disabled. Patients in the good prognosis group were more likely to recover well or to have only moderate disability (3 [11%] of 28 and 16 [57%], respectively) than were those in the poor prognosis group (0 of 26 and 7 [27%], respectively).

## Discussion

In this study, using prognosis-based outcomes, we found that patients with small spontaneous intracerebral hemorrhages in the basal ganglia (hematoma volume between 25 and 40 ml) experienced a good clinical outcome with conservative treatment. Among these patients, patients treated with conservative treatment appeared to have a beneficial effect on functional recovery at the 12-month follow-up compared with PSM patients treated with conventional craniotomy. Nevertheless, the two groups had no dramatic difference in their 12-month mortality rate.

Mendelow et al. found no significant difference between an early surgery group and an initial conservative treat group for patients with spontaneous ICH in the basal ganglia (16.9% and 17.7%, respectively) in STICH I [16]. This might be influenced by patients who had undergone delayed surgery in the initial conservative treatment group. Patients who crossed over to surgery were in a significantly worse condition than were those allocated to early surgery. Thus, the present study excluded patients who must be operated on because of a brain hernia or underwent delayed surgery because of deterioration of their condition to purely compare the efficacy of conventional craniotomy and conservative treatment to the fullest extent possible. PSM with age, GCS, volume, and midline shift was performed to bridge the gap of patients’ baseline characteristics between the two groups. Thus, the comparisons were meaningful despite the small numbers of patients.

The resulting assignment used a sliding dichotomy, referring to the design of the first STICH trial [23]. Because patients in this study tended to be older (median 55 years) and all had limb nerve deficits, independence achieved at home is worthwhile compared to relying on 24-h care for an older stroke victim. The Extended Glasgow Outcome Scale classifies the severe disability categories as dependent or independent at home. To achieve the outcome of moderate disability, the person must be able to take public transport and go shopping independently [24]. While this result might be expected in a patient with mild symptoms, a patient who was initially in a coma (GCS < 9) or severely hemiplegic would not be expected to achieve this degree of independence. Therefore, we decided to differentiate patients’ treatment goals based on their initial prognosis (according to a verified formula). This dichotomy based on prognosis greatly enhanced the power of the study to identify modest treatment effect.

To decrease any potential bias, we used interviewers blinded to the group assignment of the patients for the follow-up at 12 months. Patients were interviewed strictly based on the questionnaires for outcome assessment in order to be not biased by the interviewers’ perceptions.

The overall results of the study indicate that conventional craniotomy is not a good choice for minor hematomas in the basal ganglia. This result is surprising but makes sense. There are good clinical reasons for the hypothesis that craniotomy might be harmful for deep-seated intracerebral hemorrhage. The idea behind surgical evacuation of intracerebral hematomas is to reduce the mass effect, thereby reducing intracranial pressure, improving regional blood flow, and limiting the toxicity of blood-breakdown products [25]. Additionally, craniotomy provides a clear operative field and hematoma borders under a microscope.

However, craniotomy also requires a large scalp incision, which might cause secondary trauma [26]. Extensive siphoning and pulling in the basal ganglia region may injure any surrounding brain tissue that is still functional and may induce extensive damage [27]. It could be observed in our study that long-term functional recovery in the surgery group was worse than in the conservative treatment group, which indicates that the trauma of open craniotomy for small basal ganglia hemorrhages outweigh the benefits it brings. In addition, the spastic vessels are hard to visualize intraoperatively, which possibly increases the risk of postoperative rebleeding. Three patients in the surgery group suffered rebleeding in this study, while none of the patients in the conservative treatment group experienced this.

In addition, the surgery was performed under general anesthesia with tracheal intubation. Respiratory injuries and prolonged bed rest might explain the high rate of hospitalized pneumonia in the surgery group. Finally, hematoma evacuation through craniotomy has a relatively longer operative duration [28], which might increase the risk of mortality in patients, especially among older people [29]. The guidelines from the American Heart Association/American Stroke Association in 2015 had mentioned that the effectiveness of surgery is not well established for most patients with supratentorial ICH [19]. Thus, less invasive surgery such as clot evacuation with stereotactic [17] or endoscopic aspiration [30] have moved naturally onto the research agenda for small hematomas in basal ganglia. The stereotactic needle aspiration of basal ganglia hemorrhages (25–40 mm^3^) was compared to medical management alone in the randomized study of 465 patients. The result revealed that neurological outcome was better in the aspiration group in the 3-month follow-up, while there was no significant difference on mortality [17]. The Minimally Invasive Surgery Plus Recombinant Tissue-Type Plasminogen Activator for ICH Evacuation Trial II (MISTIE II) was designed to determine the safety and efficacy of minimally invasive surgery plus rtPA in the treatment of ICH. Seventy-nine surgical patients with 39 conservative patients were enrolled in this study which demonstrated a significant reduction of perihematomal edema in the evacuation group along with a trend toward better outcomes [31]. Therefore, the efficacy of minimally invasive clot evacuation is still uncertain.

It needs to be acknowledged that this study has some limitations. First, this study was a single-center retrospective case-control study with propensity score matching. Potential selective bias remained although it was greatly decreased. The small sample size of the present study could not provide robust evidence for clinical practice. The second limitation was that all operations were not performed by the same neurosurgeon, which might influence the consistency of surgical outcomes. Some high-quality, rigorous, randomized controlled trials are needed for further investigation of this topic.

## Conclusion

The present study’s results suggest that for patients with minor hematomas (25–40 ml) in the basal ganglion, it should be cautious to undertake conventional craniotomy. The open craniotomy might induce worse long-term functional outcomes than conservative treatment. The results of this study might provide a warning for surgical decision making for patients with minor basal ganglion hemorrhages, but further detailed research is still needed.

## Data Availability

The datasets used and analyzed during the current study are available from the corresponding author on reasonable request.

## Abbreviations

ICH: Intracerebral hemorrhage
RCTs: Randomized controlled trials
STICH: International Surgical Trial in Intracerebral Hemorrhage
CT: Computerized tomography
GCS: Glasgow coma score
mRS: Modified Rankin score
GOSE: Extended Glasgow Outcome Scale
IVH: Intraventricular hemorrhage
IQR: Interquartile range
SD: Standard deviation
OR: Odds ratios
PSM: Propensity score matching
